# Investigation of the rs7041 variable of vitamin D-connector protein gene relation with pancreatic involvement in patients with coronavirus disease 2019

**DOI:** 10.1590/1806-9282.20241191

**Published:** 2025-01-10

**Authors:** Vuslat Öztürk, Mehmet Ali Gül, Recai Aci, Âdem Keskin, Duygu Tozcu, Mustafa cihangiroğlu, Muhammed Taha Kaya, Mustafa Çapraz, Serbülent Yiğit, Akιn Tekcan

**Affiliations:** 1 Amasya University, Faculty of Medicine, Department of Medical Biology – Amasya, Turkey.; 2 Amasya University, Faculty of Medicine, Medical Biochemistry – Amasya, Turkey.; 3 Aydιn Adnan Menderes University, Söke Vocational School of Health Services – Aydιn, Turkey.; 4 Aydιn Adnan Menderes University – Aydιn, Turkey.; 5 Amasya Unιversιty, Faculty of Medιcιne, Department of Basιc Medιcal Scιences, Department of Physιology – Amasya, Turkey.; 6 Amasya University, Faculty of Medicine, Department of Infection Diseases and Clinical Microbiology – Amasya, Turkey.; 7 Ondokuz Mayιs University, Faculty of Veterinary, Department of Pathology – Samsun, Turkey.; 8 Amasya University, Faculty of Medicine, Department of Internal Medicine – Amasya, Turkey.; 9 Ondokuz Mayιs University, Faculty of Veterinary Medicine, Department of Veterinary Genetics – Samsun, Turkey.

**Keywords:** COVID-19, Pancreas, Vitamin D, Vitamin D binding protein, SNP

## Abstract

**OBJECTIVE:**

This study aims to examine whether the presence of mutation exists in the vitamin D-connector protein gene rs7041 variant of the pancreatitis table for patients diagnosed with coronavirus disease 2019.

**METHODS:**

A total of 113 patients with normal pancreatic enzyme levels diagnosed with coronavirus disease 2019 and 120 patients with both coronavirus disease 2019 diagnosis and high pancreatic enzyme levels were included in the study. The rs7041 genotyping of the 11th single nucleotide variation in the vitamin D-connector protein gene was determined by polymerase chain reaction and restriction fragment length polymorphism methods.

**RESULTS:**

In the patient group with coronavirus disease 2019 diagnosis and high pancreatic enzyme levels, the vitamin D-connector protein gene rs7041 variance GG genotype ratio was determined to be higher than the normal coronavirus disease 2019 patients. As a result of comparisons between the two groups, the difference between the genotype ratios in the relationship was determined to be statistically significant (p=0.004).

**CONCLUSION:**

Coronavirus disease 2019 patients were determined that the rs7041 halves in the vitamin D-connector protein gene could be prone to pancreatitis formation. Different populations and work with more patient groups are needed to verify the results of the study.

## INTRODUCTION

The severe acute respiratory syndrome coronavirus 2 (SARS-CoV-2) is the causative agent of the current coronavirus disease 2019 (COVID-19) pandemic, leading to over 565 million cases and approximately six million fatalities across more than 200 nations globally^1^. COVID-19 was first viewed solely as a respiratory illness. Nevertheless, the gastrointestinal system could also play a significant role, with occurrences varying between 3 and 79%^2^. Instances of increased pancreatic enzyme levels and acute pancreatitis have been linked to SARS-CoV-2, yet the underlying mechanisms causing pancreatic damage remain debated. SARS-CoV-2 employs angiotensin-converting enzyme 2 receptors alongside TMPRSS2 to penetrate and activate human cells^3^. These proteins are significantly present in both gastrointestinal luminal cells and pancreatic ductal, acinar, and islet cells^4^. Consequently, the virus has the potential to migrate from the duodenum to the pancreatic duct, subsequently affecting acinar and islet cells. This could lead to infection of the gland through a cytolytic mechanism that triggers the release of pancreatic amylases and/or lipases^5^. Additionally, an increase in pancreatic enzymes may also be attributed to kidney dysfunction. The kidneys are essential for eliminating amylases and lipases from circulation; thus, even temporary renal impairment could result in elevated levels of these enzymes.

Vitamin D is a fat-soluble hormone synthesized in the skin when exposed to UVB rays from sunlight^6^. Vitamin D is a steroid hormone that regulates calcium balance and bone mineralization in the body, working alongside parathyroid hormone^7^. Vitamin D-binding protein (VDBP) is essential for vitamin D balance, as it regulates the hormone’s half-life in circulation^8^. VDBP transports serum 25(OH)D3 into cells for vitamin D functions like immune response and apoptosis^9^. The VDBP gene is located on chromosome 4q11-q13. Two known single nucleotide polymorphisms (SNPs) have been identified in exon 11 of the VDBP gene, rs4588 and rs7041^10^. The GAT→GAG substitution in rs7041 leads to the conversion of aspartic acid to glutamic acid, and the ACG→AAG substitution in rs4588 leads to amino acid threonine substitutions for Lysine^11^. These mutations reduce vitamin D binding and may link to autoimmune disease pathophysiology. VDBP gene mutations are linked to various conditions, including thalassemia and coronary artery disease^12^.

In this study, we investigated whether the occurrence of pancreatitis in patients diagnosed with COVID-19 is associated with the rs7041 variant of the VDBP gene. The results obtained are targeted as an important step to understand the underlying cause of coronavirus-associated pancreatitis and to shape the treatment.

## METHODS

This cross-sectional study was conducted on 233 patients who were admitted to Amasya University Sabuncuoğlu Şerefeddin Training and Research Hospital, Turkey, and diagnosed with COVID-19. A G*Power statistical analysis program was used to calculate the sample size for the patient and control groups, based on ratios from previous studies with related genes. An effect size of 0.314, alpha error of 0.05, 1-beta of 0.95, and df of 5 were used, determining a minimum total sample size of 200, with at least 100 in each group^13^. After evaluating clinical data on pancreatic enzyme parameters and pancreatic involvement in COVID-19 patients, 120 patients with pancreatic involvement were grouped as the patient group, and 113 patients without pancreatic involvement were grouped as the control group. Inclusion criteria were as follows: ≥18 years old, absence of significant medical records or systemic disease, and a confirmed COVID-19 diagnosis via real-time polymerase chain reaction (PCR), lung tomography, and clinical symptoms. Additional inclusion criteria for the patient group included a diagnosis of acute pancreatitis based on at least two of the following: abdominal pain radiating to the back, serum amylase and/or lipase levels three times the upper limit of normal, and characteristic abdominal imaging findings^14^. Investigations were carried out on peripheral blood samples taken from the patient and control groups for routine biochemical examinations and included the determination of amylase, lipase, ferritin, and calcium levels. DNA was extracted from frozen blood samples previously collected for routine examinations in the biochemistry laboratory, followed by polymerase chain reaction and restriction fragment length polymorphism techniques, respectively.

### Polymerase chain reaction and genotype monitoring phase

**Figure 1:**
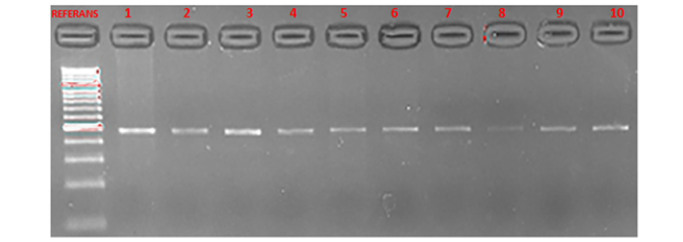
Agarose gel image after the polymerase chain reaction process.

**Figure 2.**
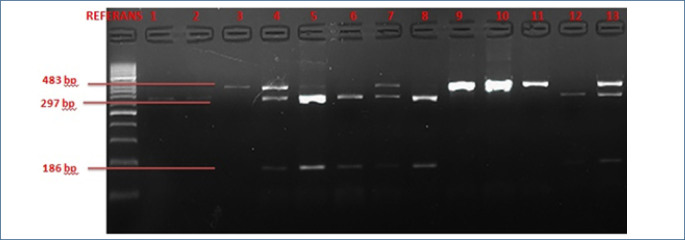
After restriction fragment length polymorphism analysis, agarose gel results showed three scenarios: One band at 483 bp indicated wild-type alleles (Asp/Asp); three bands at 483, 297, and 186 bp suggested incomplete digestion with a heterozygous allele (Asp/Glu); and two bands at 297 and 186 bp confirmed complete digestion, indicating a homozygous mutant genotype (Glu/Glu).

VDBP gene rs7041 primer sequences used in the replication of polymorphisms are as follows:

Forward 5'-AAATAATGAGCAAATGAAAGAAGAAGAC-3', Reverse 5'-TCTACTCATTTCTTGCTGTGTTTATTG-3'. PCR protocol: 1 cycle at 94°C for 5 min denaturation, 36 cycles at 94°C for 45 s denaturation, 36 cycles at 51°C for 45 s primer binding, 36 cycles at 72°C for 45 s elongation, and finally 1 cycle at 72°C for 7 min completion. After amplification, 5 μL PCR product, 0.5 μL Styl restriction enzyme, 5 μL buffer, and 9.5 μL sterile water mixture were kept at 37°C for 12 h for cutting. Three genotypes—GG (483 bp), TG (483, 297, and 186 bp), and TT (297 and 186 bp)—were expected to be formed after cutting. After cutting, the bands of the PCR products belonging to the gene regions were visualized by electrophoresis on a 2% agarose gel containing ethidium bromide. Agarose gel images of the samples after polymerase chain reaction and restriction fragment length polymorphism (PCR-RFLP) are shown in Figures 1 and 2.

### Statistical analysis

All data from patients and controls were analyzed using Statistical Package for the Social Sciences (SPSS) 21.0 and OpenEpi 3.01. Normality distribution was assessed with the Shapiro-Wilk test. Continuous data were presented as mean±standard deviation for normally distributed data and as median with interquartile ranges for non-normally distributed data. Categorical data were described with percentage frequencies. Comparisons were made using the independent sample t-test for normally distributed continuous data and the Mann-Whitney U test for non-normally distributed data. Categorical data were compared using the chi-quare (χ^2^) test. The chi-square test also assessed the genotype and allele distribution of the VDBP gene rs7041 variant. Odds ratios (OR) and 95% confidence intervals (CIs) were calculated for genotype comparisons between patient and control groups. Hardy-Weinberg equilibrium was tested for both groups. A significance level of 0.05 was used for all hypothesis testing.

## RESULTS

Descriptive and clinical data of the patients are given in Table 1. The patient group is characterized by higher age, predominance of males, and increased amylase, lipase, and ferritin levels (p<0.05). However, there was no statistically significant difference in calcium levels (p=0.35).

**Table 1.** Age, gender, amylase, lipase, ferritin, and calcium values of coronavirus disease 2019 patient and control group.

Features	Patient group n=120 (%)	Control group n=113 (%)	p
Age (mean±SD)	64.73±15.32	57.55±16.36	0.002^a^
Gender, female/male, n (%)	46/74 (38.3/61.66)	75/38 (66.38/33.62)	<0.001^b^
Amylase (U/L) median [Q1–Q3]	97.20 [73.48–114.84]	54.45 [45.70–66.30]	<0.001^c^
Lipase (U/L) median [Q1–Q3]	62.25 [45.13–81.56]	30.80 [23.38–37.95]	<0.001^c^
Ferritin (mg/L) median [Q1–Q3]	289.55 [120.41–659.98]	98.35 [56.75–175.93]	<0.001^c^
Calcium (mg/dL) (mean±SD)	8.60±0.6	8.69±0.82	0.35^a^

^a^t-tests

^b^Chi-square (χ2) test

^c^Mann-Whitney U test, SD: standard deviation.

Analysis of VDBP genotypes and alleles between patients (n=120) and controls (n=113), revealed significant differences (Table 2). The G/G genotype was more frequent in the patient group with pancreatic involvement, but the control group is characterized by predominance of the T/G and T/T genotypes. Similarly, the combined T/T+T/G genotype showed a higher frequency in controls than in patients with an OR of 1.99 (p=0.04), and the G/G genotype was more frequently observed in the patient group with an OR of 2.44 (p=0.001). Additionally, the G allele was significantly more frequent in patients (65.84%) than in controls (50.44%) with an OR of 1.89 (p<0.001). These findings suggest a strong association between VDBP genotypes/alleles and the patient group.

**Table 2.** Distribution of vitamin D-connector protein gene rs7041 variant genotype and allele frequencies according to patient and control grouping of coronavirus disease 2019 patients.

Gene	Patient n=120 (%)	Control n=113 (%)	p	OR (95%CI)
VDBP				
Genotypes				
G/G	55 (45.8%)	29 (25.7%)		
T/G	48 (40.0%)	56 (49.6%)	**0.004**	
T/T	17 (14.2%)	28 (24.8%)		
T/T/G+T/G:T/T	103:17	85:28	**0.040**	1.99 (1.02–3.94)
G/G: T/G/G+T/T	55:65	29:84	**0.001**	2.44 (1.40–4.28)
Alleles				
G	158 (65.84%)	114 (50.44%)	**<0.001**	1.89 (1.30–2.75)
T	82 (34.17%)	112 (49.56%)		

Statistically significant values are shown in bold. OR: odds ratio; VDBP: vitamin D-connector protein.

## DISCUSSION

The pathophysiology of chronic pancreatitis is highly complex and includes acinar cell damage, acinar stress responses, duct dysfunction, persistent or altered inflammation, and/or neuro-immune involvement, but these mechanisms are not fully understood. Pancreatic gland dysfunction (mainly due to loss of islets of Langerhans) is observed with exocrine pancreatic insufficiency (lack of digestive enzymes produced by the pancreas, resulting in impaired digestion) and increased risk of pancreatic ductal adenocarcinoma^14^. Acute pancreatitis is an inflammatory condition with a high complication and mortality rate. Pancreatic acinar cells are crucial in its development. Factors like gallstones or alcohol activate these cells, leading to premature activation of digestive enzymes such as trypsinogen and procarboxypeptidase A1. An alanine aminotransferase level above 150 IU/L during an attack suggests a biliary cause^15^. Following uptake or synthesis in the skin, lipophilic inactive vitamin D is reversibly bound to Vitamin D Binding Protein and, to a lesser extent, albumin in the systemic circulation. It is then transported to the liver, where it undergoes enzymatic conversion to 25(OH)D^16^. One of the main reasons why it is considered an important factor for regulating vitamin D homeostasis is that VDBP regulates the half-life of vitamin D in the systemic circulation^8^. The VDBP gene is located on chromosome 4q11-q13 and has two known SNPs rs4588 and rs7041, and three isotypes (DBP1F, DBP1S, and DBP2) in exon 11^17^.

The VDBP gene is a precursor to many other diseases. In addition, the mutation in the gene is thought to cause the development of pancreatitis in individuals with coronavirus disease, and studies are being conducted^16^. Vitamin D binds specifically to VDBP. Polymorphisms in the VDBP gene can alter the balance of circulating Vitamin D levels and its activity in the body^16^.

The role of VDBP gene polymorphisms, VDBP levels, and vitamin D levels in the mortality of sepsis patients was examined. Polymorphisms in the VDBP gene at locus rs7041 were associated with decreased VDBP and vitamin D levels, which were associated with higher mortality in sepsis patients^18^.

A study found that the GT genotype at the rs7041 locus is positively correlated with COVID-19 prevalence and mortality, while the TT genotype shows a negative correlation^19^. In our study, the 57904 T>G mutation located between 416 and 483 bp for the rs7041 variant was found to be statistically significant (p=0.004). In a study on vitamin D binding protein and pancreatic cancer, no association was found between the VDBP gene and pancreatic cancer^20^. In a study investigating Vitamin D binding protein and cancer risk, a meta-analysis of Gc polymorphisms rs2282679, rs7041, and rs4588 showed no significant correlation with pancreatic cancer^21^. Research shows that vitamin D levels can affect cancer immunotherapy. For example, 25-hydroxyvitamin D may affect the concentration of an immunotherapy drug such as nivolumab^22^. A study examined the link between high amylase and lipase levels and COVID-19 severity in patients with comorbidities. Of 1,378 individuals, 687 (49.9%) had mild infections and 691 (50.1%) had severe infections. Elevated amylase levels were found in 316 patients (23%)^23^. In these patients, amylase levels increased one to threefold in 261 patients and threefold in 55 patients. According to the Atlanta criteria, pancreatitis was found in only six of these patients (1.89%)^23^. A study found elevated serum amylase and/or lipase levels in COVID-19 patients. In Wuhan, among 121 confirmed cases, one patient (1.85%) with mild COVID-19 showed increased enzyme levels. For severe cases, 12 (17.91%) had elevated amylase and 11 (16.41%) had elevated lipase^24^. In a study including 50 different hospitals in Spain, acute pancreatitis was observed in 63,822 patients who presented to the emergency department due to coronavirus infection. The incidence of pancreatitis in patients with COVID-19 was determined to be 0.07% in this study^25^. The mean age of patients with coronavirus and pancreatitis was 64.73±15.32 years. Lipase levels were significantly higher in this group (80.86±99.21) compared to controls (31.49±9.65). Amylase values also exceeded average levels, measuring 119.31±171.65 for those with both conditions versus 56.14±14.99 for those with only coronavirus disease. Additionally, a positive correlation was found between VDBP gene polymorphism at the rs7041 locus and the prevalence of pancreatitis in COVID-19 patients.

The limitation of this study is that it was conducted in a single center. In order to obtain results that can be applied to a wider population, studies should be conducted in more than one center and with larger sample sizes.

## CONCLUSION

Our study found the T>G mutation in the VDBP gene (rs7041 variant) in COVID-19 patients with elevated pancreatic enzyme levels. Other research indicates that pancreatitis occurs in COVID-19 patients with this mutation. However, we did not detect the T>G mutation in COVID-19 patients who did not develop pancreatitis.
